# fMRI study of the role of glutamate NMDA receptor in the olfactory processing in monkeys

**DOI:** 10.1371/journal.pone.0198395

**Published:** 2018-06-05

**Authors:** Fuqiang Zhao, Marie A. Holahan, Xiaohai Wang, Jason M. Uslaner, Andrea K. Houghton, Jeffrey L. Evelhoch, Christopher T. Winkelmann, Catherine D. G. Hines

**Affiliations:** Merck & Co., Inc., West Point, Pennsylvania, United States of America; Universite de Lyon, FRANCE

## Abstract

Studies in rodents show that olfactory processing in the principal neurons of olfactory bulb (OB) and piriform cortex (PC) is controlled by local inhibitory interneurons, and glutamate NMDA receptor plays a role in this inhibitory control. It is not clear if findings from studies in rodents translate to olfactory processing in nonhuman primates (NHPs). In this study, the effect of the glutamate NMDA receptor antagonist MK801 on odorant-induced olfactory responses in the OB and PC of anesthetized NHPs (rhesus monkeys) was investigated by cerebral blood volume (CBV) fMRI. Isoamyl-acetate was used as the odor stimulant. For each NHP, sixty fMRI measurements were made during a 4-h period, with each 4-min measurement consisting of a 1-min baseline period, a 1-min odor stimulation period, and a 2-min recovery period. MK801 (0.3 mg/kg) was intravenously delivered 1 hour after starting fMRI. Before MK801 injection, olfactory fMRI activations were observed only in the OB, not in the PC. After MK801 injection, olfactory fMRI activations in the OB increased, and robust olfactory fMRI activations were observed in the PC. The data indicate that MK801 enhances the olfactory responses in both the OB and PC. The enhancement effects of MK801 are most likely from its blockage of NMDA receptors on local inhibitory interneurons and the attenuation of the inhibition onto principal neurons. This study suggests that the mechanism of local inhibitory control of principal neurons in the OB and PC derived from studies in rodents translates to NHPs.

## Introduction

The olfactory processing pathway in mammals begins at olfactory receptor neurons (ORNs) in nasal epithelium. Axons of ORNs project into olfactory bulb (OB) where they synapse with mitral cells. Mitral cells are the principal neurons in the OB and they send excitatory projections to the principal neurons (pyramidal cells) in the piriform cortex (PC) [1]. The principal neurons in the OB and PC also synapse with the local inhibitory interneurons. In the OB, granule cells are interneurons, and they form reciprocal dendrodendritic synapses with mitral cells, and inhibit mitral cells through the dendrodendritic inhibition [2, 3], where activation of NMDA receptors on granule cells is essential [4–6]. In the PC, the pyramidal cells receive inhibition from the local interneurons [7, 8], and the glutamate NMDA system is involved in the inhibition [9] [10].

Because of the inhibitions on the principal neurons from local inhibitory neurons, odorant-induced olfactory responses in the principal neurons of the OB and PC depend on not only their excitatory inputs, but also the inhibitory inputs from local inhibitory interneurons. Studies from rodents show that glutamate NMDA receptor antagonists MK801 (Dizocilpine) or APV (2-amino-5-phosphono-valerate) can enhance the olfactory responses in the principal neurons by reducing the interneuron-induced inhibition on principal neurons. This reduction of the inhibition is from the blockage of the NMDA receptor-mediated transmission from the principal neurons to interneurons [3, 4, 9–11]. However, all previous studies have been performed in rodents, and it is not clear if such findings regarding the inhibitory control of the principal neurons observed in rodents translate to humans. Since MK801 or APV cannot be directly tested in humans, nonhuman primates (NHPs) studies need to be done with the NMDA receptor antagonists to bridge the gap in understanding the role of glutamate NMDA receptor in the olfactory processing between rodents and humans. NHPs are closer to humans than rodents for olfactory processing. Anatomically, the distributions of the olfactory structures (i.e., OB and olfactory tract) on the ventral surface of brain are similar between humans and NHPs [12, 13]. The size of OB relative to total brain volume in NHPs is closer to the relative size of OB in humans than that in rodents. The relative size rather than the absolute size of the OB has been suggested to be more relevant to the olfactory capabilities for different species [14]. Functionally, based on the behavioral studies, the olfactory sensitivity of NHPs is closer to humans than rodents [15, 16].

Even though NHPs have played a vital role in understanding the neural processing in humans, the olfactory processing in NHPs, especially the effects of pharmacological agents on the olfactory processing in NHPs have not been well studied. Techniques which can measure the olfactory response in the olfactory system of NHPs are limited. An fMRI technique which can measure the olfactory response in NHPs has been successfully developed recently, and the odorant-induced olfactory fMRI activations can be robustly observed in the OB of NHPs [17]. This fMRI technique can be used to study the mechanism of olfactory processing in NHPs by testing the effects of pharmacological agents on the odorant-induced olfactory fMRI signals.

MK801 has been used to block NMDA receptor *in vivo* in rats [4, 11, 18] and NHPs [19, 20]. In rodents, MK801 enhances the odorant-induced neural activations in the OB and PC by blocking the interneurons-induced inhibition onto principal neurons [4, 11, 21]. If the inhibitory control of principal neurons mechanism in the olfactory structures of NHPs is similar to that in rodents, it is expected that MK801 would also enhance the odorant-induced olfactory responses in the OB and PC of NHPs.

Cerebral blood volume (CBV) fMRI, which uses an intravascular contrast agent consisting of ultrasmall superparamagnetic iron oxide nanoparticles (USPIO), has been used to study odorant-evoked olfaction in rats [22, 23], and NHPs [17]. CBV fMRI is sensitive to neural activation-induced vascular responses, and has a higher sensitivity compared to BOLD fMRI [24, 25]. A previous high-resolution CBV fMRI study in the OB of rats showed that neural activities in both principal neurons and interneurons induce vascular dilation (CBV increase), but odorant-induced increases of CBV are dominated by neural activities in the principal neurons [22].

In this study, CBV fMRI was used to study the effects of MK801 on the odorant-induced olfactory processing in the OB and PC of NHPs to determine the role of glutamate NMDA receptors in the olfactory processing in NHPs.

## Materials and methods

### Animal preparation

All procedures were performed in accordance with our institution's (Merck & Co., Inc.) Institutional Animal Care and Use Committee (IACUC) guidelines at the Merck Research Laboratories facility, which is AAALAC-accredited (AAALAC: The Association for Assessment and Accreditation of Laboratory Animal Care International). The studies were conducted in full compliance with the Guide for the Care and Use of Animals, 8th edition (National Research Council-US, 2011). Four normal female monkeys (Macaca mulatta) were used on this study. The monkeys were 5–7 years of age (weight range: 5.5 to 8.3 kg (6.4 ± 0.9 kg, mean ± std). All animals were purpose-bred from an IACUC approved vendor source, and housed in a controlled room environment (temperature 72 ± 3°F, humidity 40%–60%, 12 hour light-12 hour dark cycle with fluorescent lighting, 10–15 air changes per hour). The temperature and relative humidity were monitored continuously. Monkeys were fed Certified Monkey Diet® #5045 (Purina Mills, Inc Richmond, IN) once a day as well as fruits and vegetables, and given water ad libitum using lixits. The water provided received no other treatment than being passed through a 5 μm filter.

Each animal was initially anesthetized using ketamine (3 mg/kg, i.m.) and dexmedetomidine (40 μg/kg, i.m.) for catheterization and experiment set-up. Two intravenous catheters were implanted into both saphenous veins for fluid support, and deliveries of dexmedetomidine, contrast agent and MK801. Each animal was then secured in the supine position in the MRI scanner, and the anesthesia changed to a continuous delivery of isoflurane (0.25%) and dexmedetomidine (i.v. infusion of 15 μg/kg/hr). During the entire experiment session, the animal was kept under spontaneous respiration. Oxygen-enriched gas (N_2_:O_2_ = 3:2) was used to maintain the blood oxygen saturation above 96%. The gas was delivered via Silastic tubing (0.078" ID x 0.125" OD) to nostrils. Body temperature was measured by a probe placed in the axilla and maintained by a circulating warm-water blanket. The respiration was monitored by a pressure sensor connected to a balloon secured to the chest. Blood oxygen saturation and heart rate were monitored by an optical probe attached to a toe. At the end of the experiments, atipamezole HCl (0.25 mg/kg, i.m.) was administered to reverse dexmedetomidine anesthesia.

The USPIO contrast agent (Feraheme, ferumoxytol injection, AMAG pharmaceuticals, Cambridge, MA) was administered (10 mg/kg, i.v.) before the start of fMRI data acquisition. A preliminary study showed that the Feraheme has a half-life time of 12.12 ± 3.05 hrs (mean ± std, n = 6) in NHPs, which is similar to the Feraheme half-life time of >15 hours reported in the human CBV fMRI study (Srihasam et al., 2010). To maintain a relatively constant concentration of the agent in blood, the USPIO was continuously i.v. infused at the rate of 0.41 mg Fe/kg/h after the initial bolus.

### Odor stimulus

Olfaction was induced with the odorant isoamyl-acetate. A gas mixture (2 liter/min medical air + 0.8 liter/min O_2,_ with or without odor) was constantly delivered by two small tubes (Silastic tubing, 0.078" ID x 0.125" OD), which were loosely placed in the nostrils at the depth of ~1 cm (Note: Teflon or Polypropylene tubing would be better than Silastic tubing because they have less odor sorption based on Dr. Yevgeniy Sirotin’s study. See http://mylabtime.blogspot.com/2012/04/tubing-and-odors-non-ideal-combination.html). The gas flow was split into two pathways, one of which received odorized gas through a bubbling bottle containing the odor solution. The two pathways join together prior to entering the nostrils [17]. During the odor stimulation period, the odor was introduced by opening two valves at the inlet and outlet of the bubbling bottle through a control signal from MRI sequence. The concentration of isoamyl-acetate in the gas delivered was estimated to be ~2870 ppm [26].

### MRI measurement

**Fig 1:**
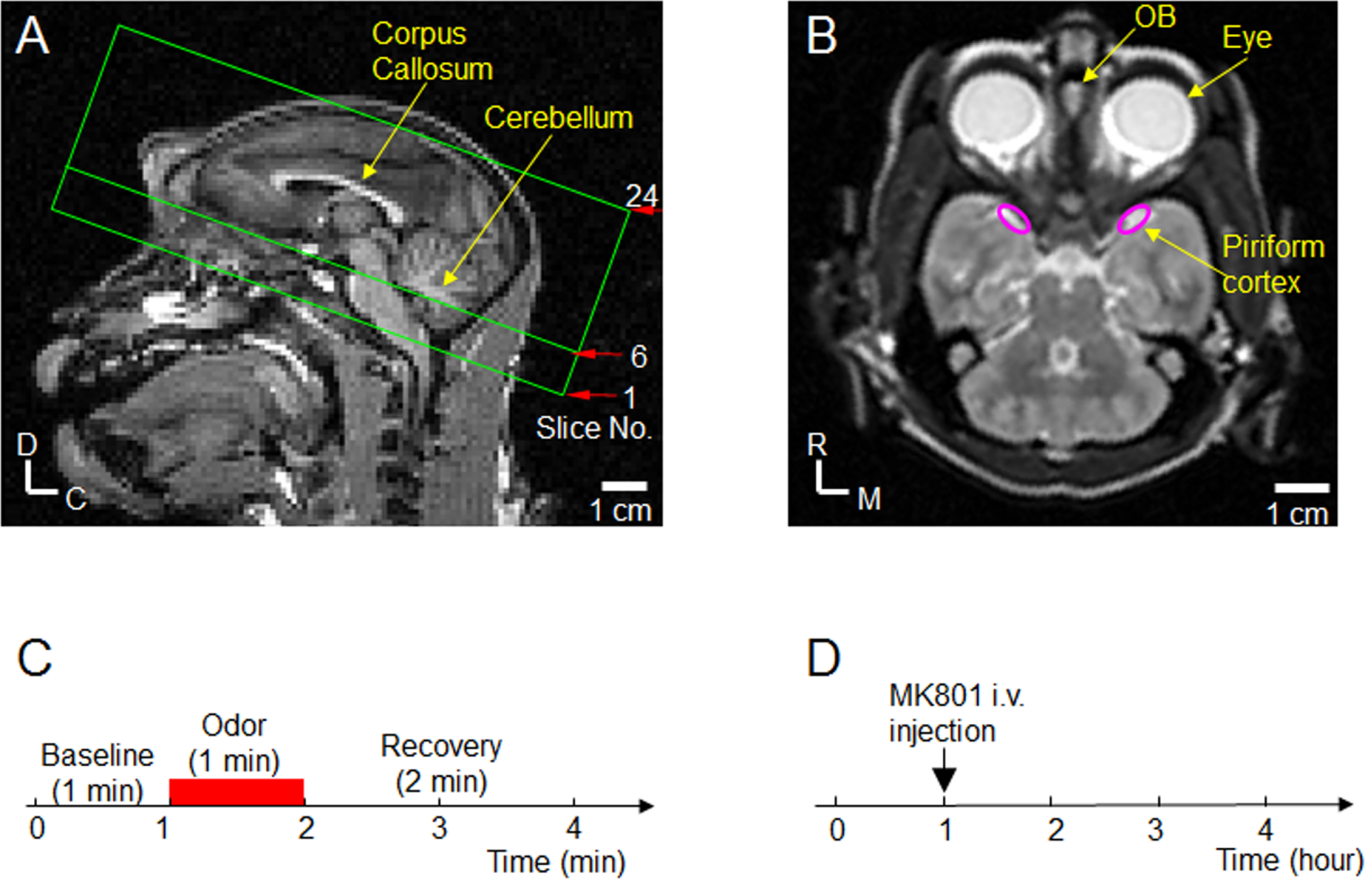
fMRI slice selection and study design. (**A**) T_1_-weighted sagittal anatomical image was used for fMRI slice selection. Locations of twenty-four consecutive axial slices chosen for the functional study are outlined by green lines. The direction of the slices is parallel to the corpus callosum. D: Dorsal; C: Caudal. (**B**) T2 axial image corresponding to the slice No. 6 in (A) shows the well-identifiable olfactory bulb (OB). Based on Fig 12 in [27], locations of the bilateral piriform cortices are outlined by the two pink ovals. R: Rostral; M: Medial. (**C**) Odor stimulation paradigm. Each 4-min fMRI measurement includes 1-min baseline, 1-min odor delivery (red bar), followed by 2-min recovery. For each animal, sixty fMRI measurements were performed during a 4-h period. (**D**) MK801 study design. MK801 was intravenous injected at 1-h of the 4-h experimental period. Fifteen fMRI measurements were made before MK801 injection, and forty-five fMRI measurements were made after MK801 injection.

MRI measurements were performed on a 3-T, Siemens TRIO (Siemens, Erlangen, Germany). A 16-channel head coil was used as the radiofrequency (RF) receiver. T1-weighted anatomical images of sagittal slices were obtained using three-dimension (3D) magnetization-prepared rapid gradient-echo (MPRAGE) sequence: TI (inversion time) = 0.9 s, TR (repetition time) for each segment = 2.3 s, TE = 3.3 ms, matrix size = 128 × 128, field of view = 15 × 15 cm^2^, slice thickness = 1.8 mm. Based on the center sagittal T1-weighted image, twenty-four consecutive axial slices parallel to the corpus callosum were chosen (Fig 1A) for fMRI data acquisition. Fig 1B shows the T2 axial image corresponding to the slice No. 6 in Fig 1A in which the OB and PC are located. fMRI data acquisition started >0.5 hour after initiating the combined dexmedetomidine-isoflurane anesthesia. fMRI measurements were made during a 4 hour period for each NHP. Single-shot gradient echo echo-planar imaging (GE EPI) sequence was used for fMRI data acquisition: matrix size = 64 × 64, field of view = 12 × 12 cm^2^, slice thickness = 1.8 mm, repetition time (TR) = 3 s, and gradient echo time (TE) = 28 ms. The corresponding spatial resolution was 1.9 × 1.9 × 1.8 mm^3^, and the acquisition time for imaging the entire volume was 3 s. Each fMRI measurement required 4 min, with a total of 20 (1 min baseline) + 20 (1 min odor stimulation) + 40 (2 min recovery) volume acquisitions as shown in Fig 1C. For each NHP, sixty fMRI measurements were made during a 4-h period. MK801 was injected intravenously as a bolus 1 hour after starting the fMRI acquisition (Fig 1D). After MK801 injection, fMRI data were continuously acquired for 3 hours. Therefore, fifteen fMRI measurements were done before MK801 injection, and forty-five fMRI measurements were done after MK801 injection.

MK801 (Sigma-Aldrich) was dissolved in sterile 0.9% physiological saline at the concentration of 1 mg/ml, and the volume dose was 0.3 ml/kg. The MK801 dose of 0.3 mg/kg in this study is consistent with the dose used in the previous studies in NHPs [19, 20].

### fMRI data analyses

Data was processed using Stimulate (Strupp, 1996) and custom MATLAB routines (Version 7.12.0, Mathworks, Natick, MA). The images of the fMRI data from each animal were first realigned to template images using a maximum cross correlation with rigid body spatial transformations. The data from the 15 fMRI measurements of all animals before MK801 injection were averaged to map the olfactory activations in the naïve condition. The data from the 45 fMRI measurements of all animals after MK801 injection were averaged to map the olfactory activations to observe MK801 effects.

Based on the averaged data, statistical *t* value maps were computed by comparing the experimental fMRI data acquired during control and stimulation periods on a pixel-by-pixel basis. The control periods included the period of the 1-min baseline and the period from 1 min after cessation of odor stimulation to the end of fMRI measurement (3 − 4 min in Fig 1C); the first 1 min data after the cessation of odor (2 − 3 min in Fig 1C) was ignored because post-stimulus fMRI signals did not quickly return to pre-stimulus signal levels. Similarly, the stimulation period included the period from the 6 s after onset of stimulation to the end of the stimulation to account for any non-stable hemodynamic responses within the initial 6 s after initiating stimulus. To detect activation, statistical threshold of p<0.05 and the contiguous cluster size of 4 [28] were simultaneously used in this study [23, 29]. Based on the assumption that brain regions of true neural activity tend to induce fMRI signal changes over contiguous pixels, using Monte Carlo simulations, Forman et al. have established the per-pixel probability of detecting false positive pixels as a function of statistical threshold and cluster size for neuroimaging study [28]. The per-pixel false positive probability corresponding to the statistical threshold of p<0.05 and the cluster size of 4 is p<0.0017 (the 6th element in the 4th row of Table 1 in [28]). To show fMRI activations, color-encoded percentage fMRI signal changes were overlaid on averaged EPI images; increases in neural activity (CBV increase) were displayed with hot colors (red/yellow).

Two regions of interest (ROI) defined from the activation maps after MK801 injection were selected for further quantitative analysis: (1) the olfactory bulb consisting of the activated pixels in the olfactory bulb region, and (2) the piriform cortex consisting of the activated pixels in the regions where the piriform cortex is located.

To examine the reproducibility of the fMRI activations in the two ROIs observed after MK801 injection, the 45 fMRI measurements after MK801 injection were divided into odd and even measurements for each NHP. The data from odd and even measurements of all NHPs were then averaged separately to calculate the fMRI signal changes in the two ROIs. The reproducibility of activations was tested by evaluating the correspondence of the fMRI activations between odd and even fMRI measurements.

To examine the MK801 effect on the olfactory fMRI responses, the 4-h experiment period was divided into four 1-h periods. The time courses of fMRI signals from the 15 fMRI measurements in each 1-h period were averaged. As such, the fMRI data acquired during the 4-h experiment session was reduced to the equivalent of 4 responses. The responses in the four 1-h periods were then used to examine the statistical significance of MK801 effects on the olfactory fMRI responses. The strengths of the olfactory fMRI response during these four 1-h periods were calculated by averaging the amplitudes of fMRI signals during the 1-min stimulation period from the averaged time courses. Statistical significance was analyzed using a *t*-test by comparing the response strengths after MK801 injection with the response strength before MK801 injection.

### Statistics

Statistical significance was examined by student paired or non-paired *t*-tests (p<0.05).

## Results

### MK801 effect on physiological parameters

**Fig 2:**
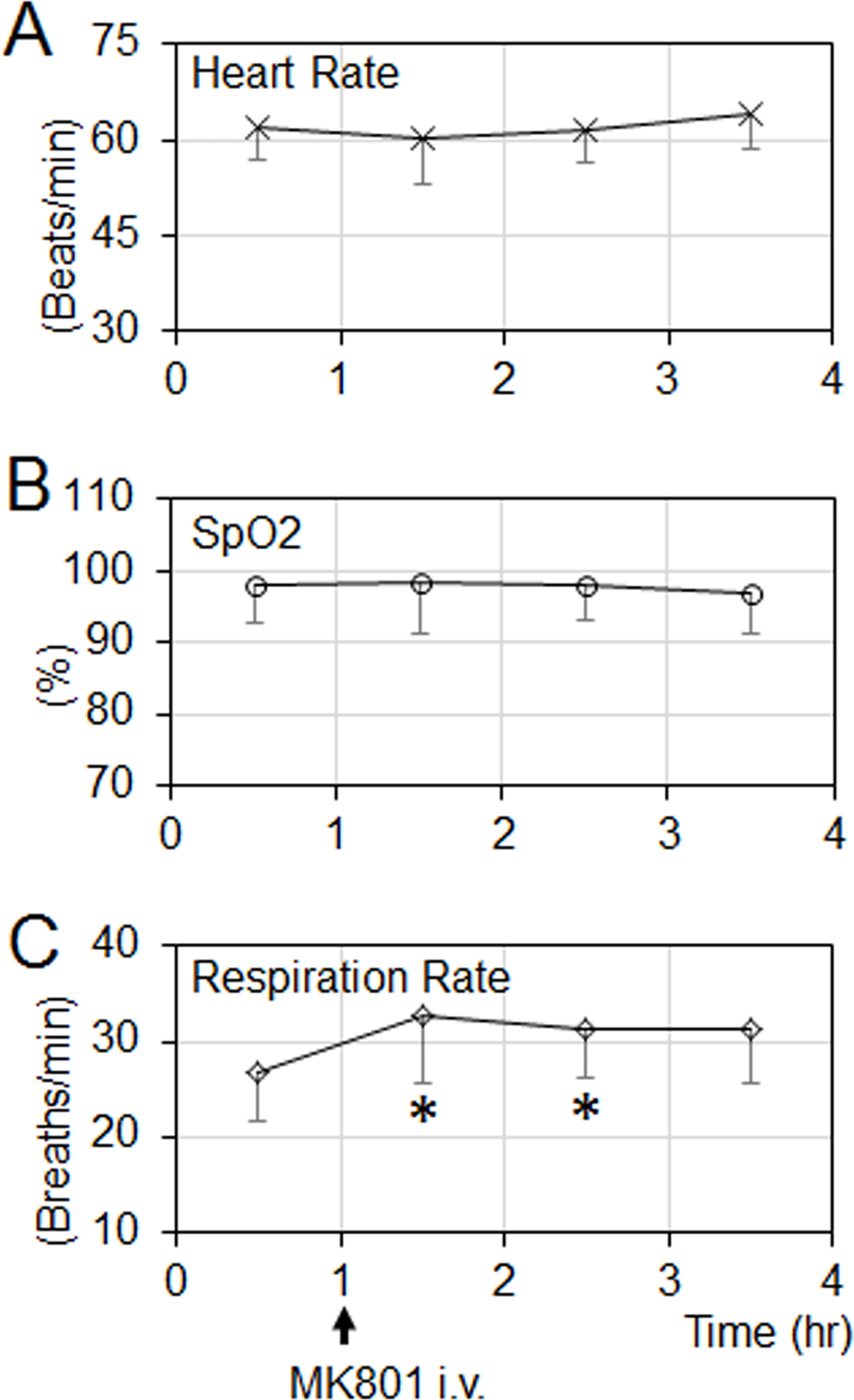
Cardiorespiratory parameters during the 4-h experimental period (mean ± SD, n = 4). (**A & B**) MK801 has no significant effect on the heart rate and blood oxygen saturation (SpO2). (**C**) MK801 significantly increased the respiration rate. The asterisks (*) indicate statistical differences comparing the respiration rate before MK801 injection (paired *t*-test, all p<0.006).

In this study, no consistent changes in heart rate and blood oxygenation saturation (SpO2) were observed after MK801 delivery (Fig 2A and 2B). However, MK801 increased the respiration rate (Fig 2C). The respiration rate increase had no significant effect on the olfactory responses (see Discussion).

### Spatial locations of olfactory fMRI activations under naïve condition

**Fig 3:**
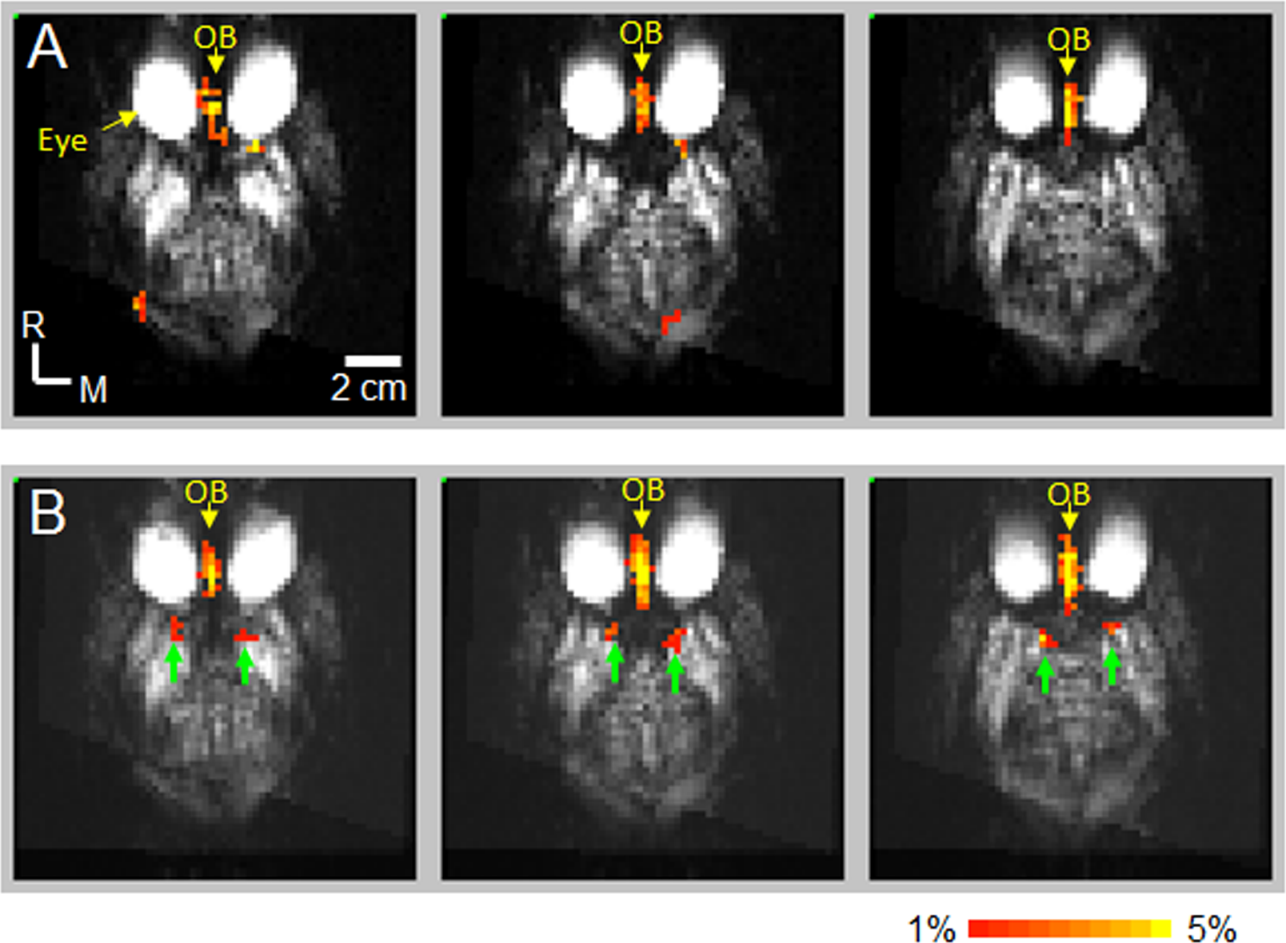
Olfactory fMRI activations before and after MK801 injection. The data from all NHPs were averaged to map the activations (p < 0.05, cluster size = 4). (**A**) Olfactory fMRI Activations *before* MK801 injection. Color-encoded activations (red/yellow) were observed in the consecutive 3 slices where olfactory bulb is located. **(B)** Olfactory fMRI activations *after* MK801 injection. Color-encoded activations were observed in the consecutive 3 slices where olfactory bulb and piriform cortex are located. Green arrows: activations in the piriform cortex. OB: olfactory bulb.

To observe the spatial locations of olfactory fMRI activations under the naïve condition, the data from the 15 fMRI measurements of all animals before MK801 injection were averaged to map the odorant-induced fMRI activations. Fig 3A shows the activation maps in the 3 consecutive axial slices in which the OB and PC are located. fMRI activations (red/yellow) were observed in the OB. Robust activations were observed only in the OB which is consistent with our previous report [17].

### MK801 effect on the spatial locations of olfactory fMRI activations

To observe MK801 effects on the spatial locations of olfactory fMRI activations, the data from the 45 fMRI measurements of all animals after MK801 injection were averaged to map the olfactory activations to observe MK801 effects. Fig 3B shows the activation maps in the 3 consecutive axial slices in which the OB and PC are located. Unlike the activation maps prior to MK801 injection, activations after MK801 injection were observed not only in the OB, but also in the locations where the PC are located.

**Fig 4:**
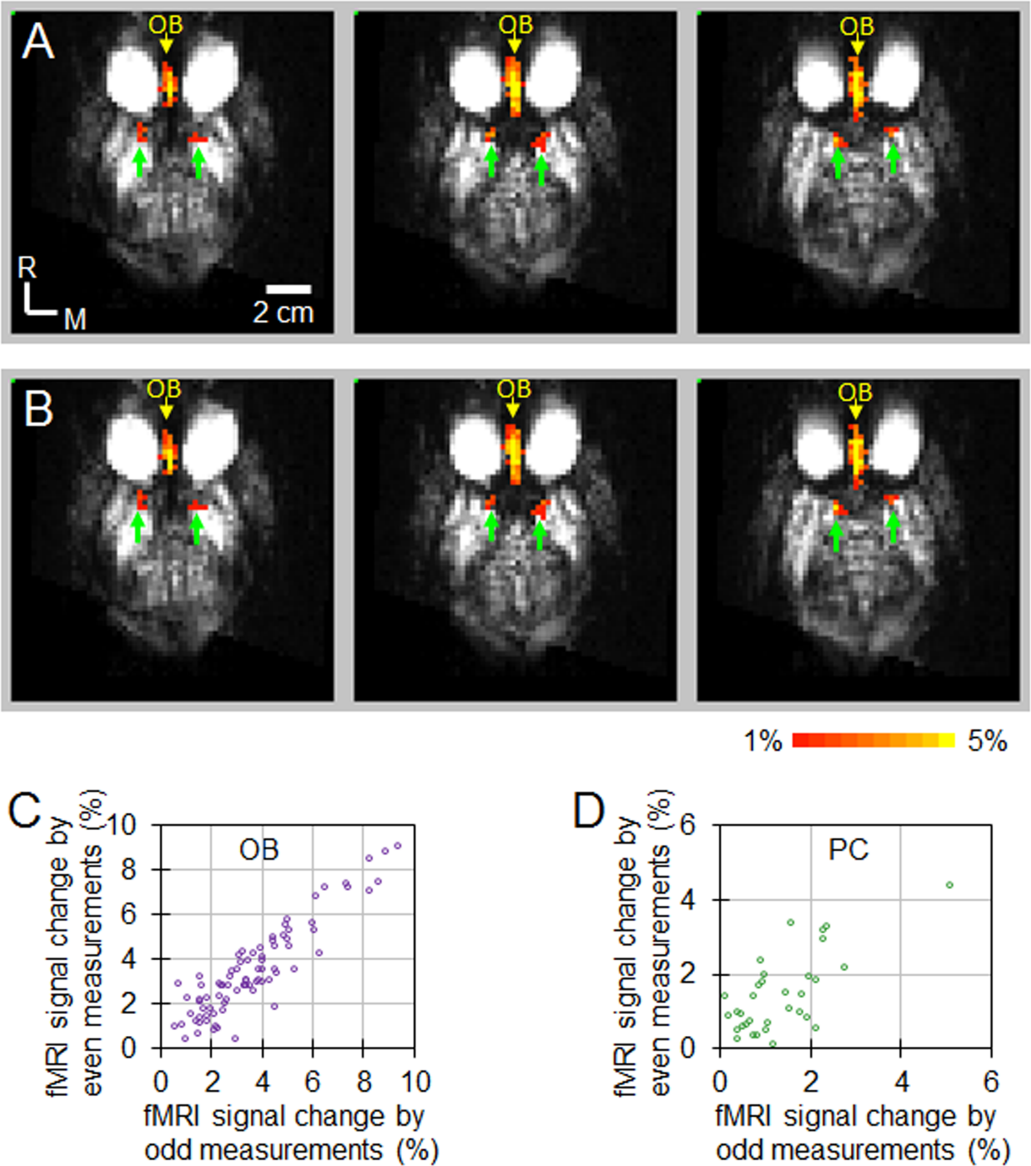
Reproducibility of olfactory fMRI activations in the OB and PC after MK801 injection. The data from all NHPs after MK801 injection were averaged to map the activations (p < 0.05, cluster size = 4) to define the ROIs of OB and PC. Within the OB and PC ROIs, color-encoded percentage fMRI signal changes were calculated from the averaged data over odd measurements (**A**) and from the averaged data over even measurements (**B**). The percentage change of fMRI signals for each voxel within the OB and PC for the two subsets are plotted against each other in (**C**) and (**D**). As shown in these figures, data points predominantly lie along their diagonal lines (slope = 1), indicating the fMRI signal changes from odd subsets are quantitatively similar to signal changes from even subsets. For the activations in OB (C), the cross correlation value is 0.91 (*p* = 1.41e^-42^). For the activations in PC (D), the cross correlation value is 0.70 (*p* = 9.65e^-7^). OB: olfactory bulb. PC: piriform cortex.

The reproducibility of these activations was tested, and the results were shown in Fig 4. The activations in the OB and PC were highly reproducible, indicating that they are true activations. Comparing the activations before MK801 injection shown in Fig 3A, MK801 increased the activations in the OB as more voxels showed activation (from 34.2 ± 6.1 voxels before MK801 injection to 54.8 ± 21.4 voxels after MK801 injection). The MK801 enhancement of the activations in the OB in the ***anesthetized*** NHPs is similar to what has been reported using c-fos mRNA in rats under ***conscious*** condition [21] [4]. In the PC, MK801 enables the activations observable by fMRI (from no activations before MK801 injection to 14.8 ± 11.6 voxels after MK801 injection), indicating that MK801 enhanced the odorant-induced responses in the PC.

### Consistency of olfactory fMRI responses and statistical significance of MK801 effect

**Fig 5:**
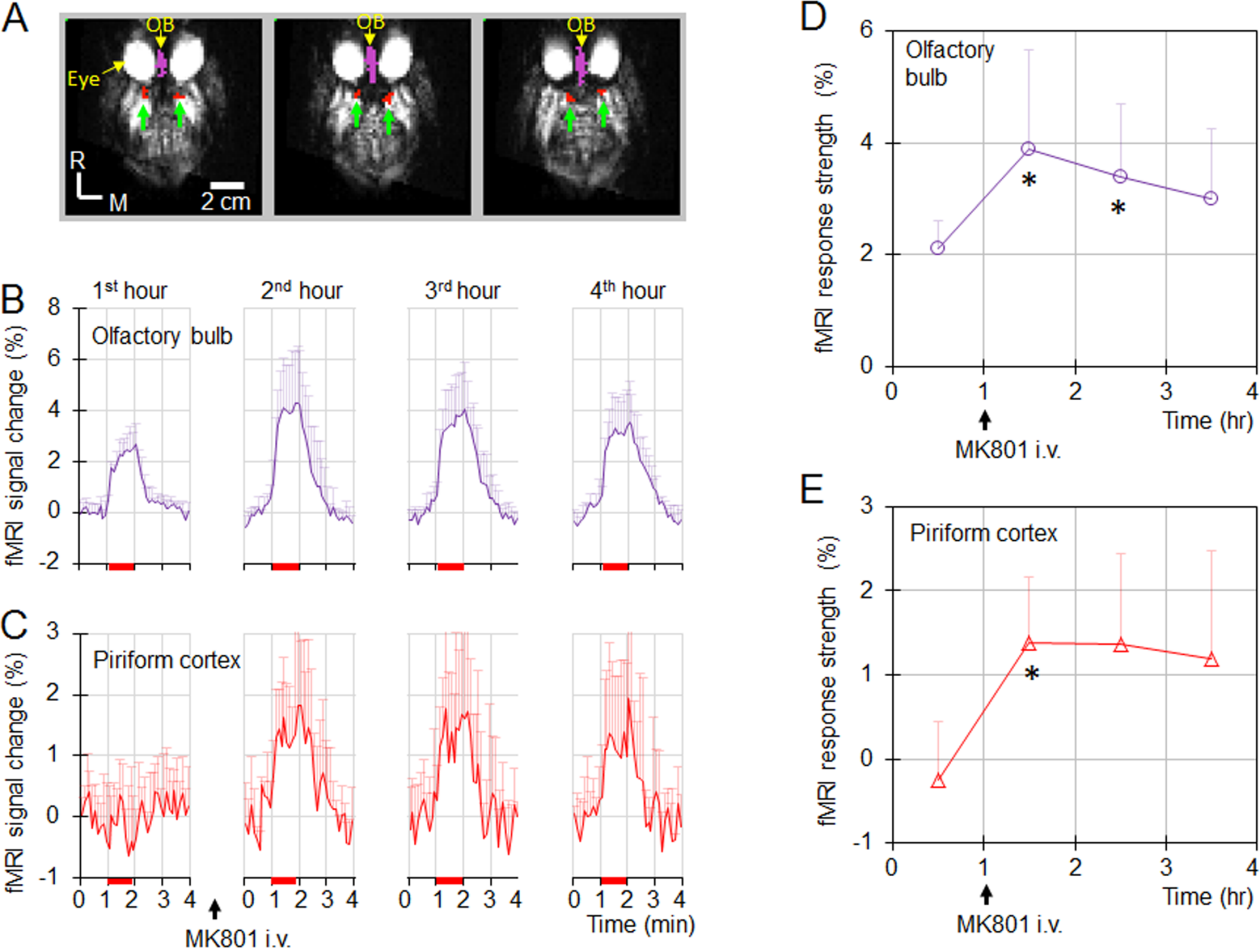
Definition of the 2 ROIs and the olfactory fMRI responses in these ROIs before and after MK801 injection. (**A**) The color-encoded ROIs were superimposed on the 3 consecutive axial gradient-echo EPI images. Purple: olfactory bulb (OB); red: piriform cortex (PC). (**B & C**) Olfactory response in the OB and PC in the different 1-h periods of 4-h experiment session (mean ± SD, n = 4). Red bars: 1-min odor stimulation. In the OB (**B**), robust olfactory fMRI response was observed before MK801 injection, and MK801 increased the response. In the PC (**C**), no robust olfactory response was observed before MK801 injection. After MK801 injection, robust and repeatable olfactory responses were observed. (**D & E**) Strengths of the olfactory responses during the 4-h experimental session (mean ± SD, n = 4). MK801 increased the response amplitudes in the OB and PC. The asterisks ‘*’ indicate that the strengths of response were significantly larger than the strengths during the first hour before MK801 delivery (paired *t*-test, all p<0.04).

To examine the consistency of olfactory fMRI responses, the 4-h experiment period was divided into four 1-h periods. The time courses of fMRI signals in the OB and PC (Fig 5A) from the 15 fMRI measurements in each 1-h period were averaged. Fig 5B and 5C show the time courses during the four 1-h periods in the OB and PC, respectively. In the OB, robust CBV responses to the odor stimulations in the OB were observed before and after MK801 injection. MK801 increased the amplitude of the CBV response. In the PC, no reliable response was observed before the MK801 injection; however, after the MK801 injection, robust and repeatable responses were observed. The consistency of the responses during the different 1-h periods further validate that the fMRI activations in the OB and PC are from true odorant-induced activations.

To analyze the statistical significance of the MK801 effect on olfaction, the mean amplitudes of the fMRI activations during the odor stimulation were calculated from the time courses shown in Fig 5B and 5C. The temporal profiles of the fMRI amplitudes in each ROI during the 4-hour period were shown in Fig 5D and 5E. MK801 increased the response amplitudes in both regions. Statistical analysis shows that the amplitudes in the first 1 hour immediately after the MK801 injection were significantly higher than the amplitudes before the MK801 injection (paired *t*-tests, all p<0.04).

## Discussion

### Summary of findings

The major findings of this study are: 1) Comparable to MK801 effect on the olfactory processing in rats [11], MK801 enhances the odorant-evoked fMRI activations in the OB and PC of NHPs, indicating that glutamate NMDA receptor plays a role in the olfactory processing in NHPs. 2) With the enhancement of MK801, the olfactory activations in the PC are measurable by fMRI.

### Respiration rate has no substantial effect on fMRI activations

**Fig 6:**
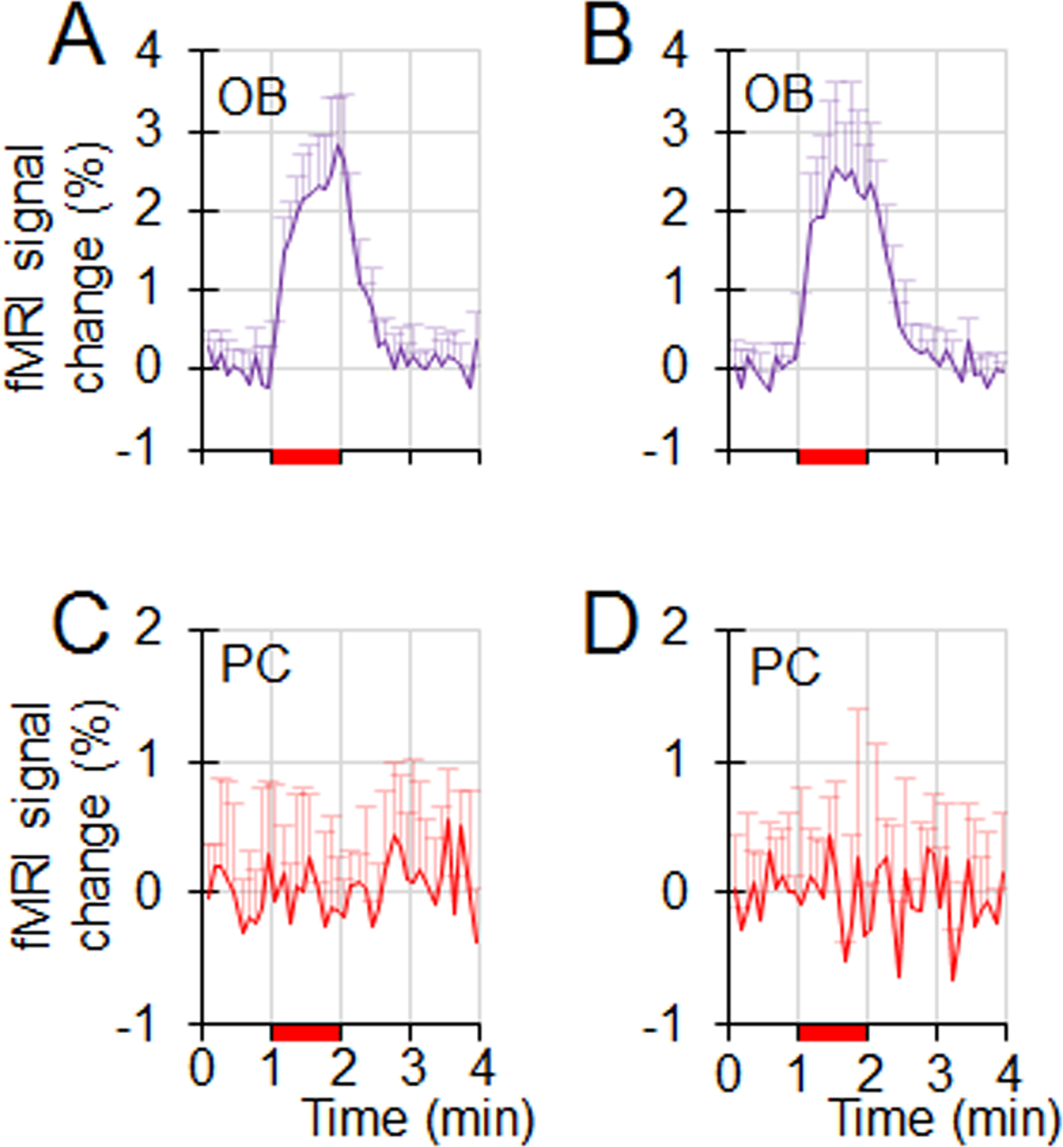
The impact of respiration rate on the olfactory responses in naïve NHPs. Data are from seven NHPs that did not receive any pharmacological agents. The 7 NHPs were separated into two groups based on their respiration rates. (**A & C**) Olfactory responses in the OB and PC from 4 NHPs with lower respiration rates of 19–25 breaths/min. (**B & D**) Olfactory responses in the OB and PC from 3 NHPs with higher respiration rates of 29–35 breaths/min. The difference in respiration rate from these 2 groups of NHPs has no effect on the olfactory responses in the OB and PC.

In this study, MK801 increased the respiration rate (Fig 2C). Respiration rate increases may elevate the olfactory response in ORNs, therefore increase the excitatory drive to the mitral cells in OB, and enhance the olfactory response in downstream structures (e.g., OB and PC). To ensure that the observed MK801 effect on the olfactory responses shown in Fig 5 is not caused by the MK801-induced respiration rate change, the olfactory fMRI data during the initial one hour period without receiving any pharmacological agents from 7 different NHPs were analyzed (4 NHPs from this study and 3 NHPs from a legacy olfactory fMRI study with identical acquisition parameters). These 7 NHPs were separated into 2 groups based on their respiration rates. Group1 contained 4 NHPs with a low respiration rate ranging from 19 to 25 breaths/min (mean ± std, 22 ± 3), while group 2 contained 3 NHPs with higher respiration rate ranging from 29 to 35 breaths/min (mean ± std, 31 ± 3). The respiration rates from these 2 groups were significantly different (non-paired t-test, *p* = 0.004), and the difference (9 breaths/min) was higher than the MK801-induced respiration rate increase (6 breaths/min). Fig 6 shows the olfactory responses in OB and PC from these two groups. In the PC, neither groups showed any response. In the OB, there was no significant difference in the response strengths (non-paired t-test, p = 0.43). Therefore, the difference in respiration rate had no significant effect on the olfactory responses from these two groups of NHPs, suggesting that the observed MK801 effect in Fig 5 was from the MK801 modulation of the olfactory responses in the OB and PC, not from the MK801-induced respiration rate increase.

### Glutamate NMDA receptor plays a role in the olfactory processing in NHPs

As indicated in Fig 5, MK801 increases the odorant-induced fMRI signals in the OB and PC of NHPs. Since the spatial resolution used in this study is low, the principal neuron-dominant regions and interneuron-dominant regions in the OB and PC cannot be separated. The observed increase of fMRI signals can be caused by the increase of neural activities in both principal neurons and inhibitory neurons. Previous high spatial resolution CBV fMRI and 2-deoxyglucose (2-DG) studies in the OB of rats show that odorant-induced increase of CBV or glucose uptake is dominated by neural activities from principal neurons (i.e., glomeruli and mitral cells), and the increase caused by interneurons (granule cells) is relatively small [22, 30, 31]. The observed MK801 enhancement of fMRI signals in this study should be mainly from the increase of neural activities in principal neurons in the OB and PC of NHPs.

In the OB, mitral cells (principal neurons) and granule cells (interneurons) form reciprocal dendrodendritic synapses [2, 3]. Odor-induced activations in mitral cells evoke glutamate release from the dendrites of the dendrodendritic synapses. The released glutamate activates the granule cells through NMDA receptors located at the dendrites of granule cells. The activations in granule cells in-turn inhibit mitral cells [3, 5]. MK801 blocks NMDA receptors in granule cells, the glutamate released from the dendrites of mitral cells is not able to activate granule cells, resulting in the stoppage of feedback inhibition onto mitral cells, and the responses in mitral cells are increased. The observed MK801 enhancement of the olfactory fMRI signals in the OB of NHPs indicates that the local inhibitory control mechanism observed in the OB of rodents translates to NHPs.

In the PC, the principal neurons (pyramidal cells) receive excitatory input from mitral cells and inhibitory input from the local inhibitory interneurons [7]. The neurotransmitter which mediates interneurons-induced inhibition of pyramidal cells is glutamate [32], and glutamate NMDA/AMPA antagonists block the interneuron-induced inhibition [9, 10]. In this study, MK801 enhances the olfactory response in the PC (Fig 5C and 5E). The enhancement is from either the increase of excitatory drive from OB and/or the decrease of the inhibition from interneurons. If the increase in the excitatory drive from OB is the major source, the enhancement in the PC should be quantitatively equal to the enhancement in OB. As shown in Fig 5, the enhancement in the PC is bigger than the enhancement in the OB, indicating the contribution to the enhancement by the decrease in the interneuron-caused inhibition. Such a decrease in inhibition is caused by MK801 blockage of the glutamate transmission from principal neurons to interneurons in the PC. The result that MK801 enhances the olfactory response in the PC of NHPs (Fig 5) suggests that the mechanism about the inhibitory control to pyramidal cells observed in rodents translates to the olfactory processing in the PC of NHPs.

### Lack of fMRI activations in piriform cortex under naïve condition

In this study, PC shows no fMRI activations without MK801 delivery (Figs 5C, 6C and 6D), which is consistent with the results from our previous report [17]. The lack of fMRI activations in naïve NHPs is most likely caused by the weak response in the PC. The characteristics of the olfactory processing in the PC [8] may contribute to the weak response. Based on studies from rodents, the characteristics of the olfactory processing in the PC are “sparse coding” and “global inhibition” [8]. Sparse coding indicates that odorant-induced activations in pyramidal cells are sparse; only a small percentage of the cells respond to the stimulation by a specific odorant. Global inhibition indicates that odorant-evoked inhibition is widespread in pyramidal cells due to the inhibition from the local interneurons which receive ubiquitous and nonselective odor-evoked excitation [8]. Because of these characteristics, the odorant stimulation induces activations only in a small number of pyramidal cells, and inhibits the spontaneous activations in widespread pyramidal cells. The fMRI signal from a voxel is the activation of all pyramidal cells within the voxel. The inhibited spontaneous activations in the pyramidal cells due to global inhibition would compensate the increased activations in the odorant-responsive pyramidal cells, minimizing the overall neural activity increase. Therefore, the sparse encoding and global inhibition make it difficult for fMRI to measure the olfactory response in PC under naïve condition.

After MK801 delivery, it interrupts the glutamate NMDA transmission to the interneurons, the NMDA receptor-mediated activations in the interneurons is blocked [9, 10, 32], and the global inhibition to pyramidal cells by interneurons is stopped. Without the global inhibition, the suppression on the spontaneous neural activities in the pyramidal cells during the stimulation period is eliminated. The olfactory fMRI response is dominated by the neural activity increases in odorant-responsive pyramidal cells, and the fMRI activations are observed in the PC as shown in Figs 3B, 5C and 5E.

### Systemic delivered MK801 has less effect on the neural transmission between principal neurons

NMDA receptor not only plays a role in the neural transmission between principal neurons and interneurons, in-vitro electrophysiology and local pharmacology studies suggest that it also plays a role in the neural transmission between the principal neurons of the different olfactory structures [7, 10, 33, 34]. If the effect of systemic delivered MK801 is dominated by the blockage of the transmission between the principal neurons, MK801 would suppress the olfaction in the principal neurons of downstream olfactory structures (e.g., mitral cells in OB and pyramidal cells in PC). Decreases in the olfactory responses of these downstream olfactory structures should be observed after MK801 injection, which are contradictory to our data as shown in Figs 3 and 5. Therefore, systemic delivered MK801 has a less effect on the neural transmission between the principal neurons in the olfactory processing pathway.

### Conclusions

We demonstrate for the first time that the odorant-induced olfactory responses in the OB and PC in NHPs are increased by MK801. The observation described in this report suggests that fMRI is a valuable tool to study the mechanism of the olfactory processing in NHPs.
